# Automatic ROI Selection in Structural Brain MRI Using SOM 3D Projection

**DOI:** 10.1371/journal.pone.0093851

**Published:** 2014-04-11

**Authors:** Andrés Ortiz, Juan M. Górriz, Javier Ramírez, Francisco J. Martinez-Murcia, for the Alzheimer's Disease Neuroimaging Initiative

**Affiliations:** 1 Communications Engineering Department, Universidad de Málaga, Málaga, Spain; 2 Department of Signal Theory, Networking and Communications, University of Granada, Granada, Spain; Indiana University, United States of America

## Abstract

This paper presents a method for selecting Regions of Interest (ROI) in brain Magnetic Resonance Imaging (MRI) for diagnostic purposes, using statistical learning and vector quantization techniques. The proposed method models the distribution of GM and WM tissues grouping the voxels belonging to each tissue in ROIs associated to a specific neurological disorder. Tissue distribution of normal and abnormal images is modelled by a Self-Organizing map (SOM), generating a set of representative prototypes, and the receptive field (RF) of each SOM prototype defines a ROI. Moreover, the proposed method computes the relative importance of each ROI by means of its discriminative power. The devised method has been assessed using 818 images from the Alzheimer's disease Neuroimaging Initiative (ADNI) which were previously segmented through Statistical Parametric Mapping (SPM). The proposed algorithm was used over these images to parcel ROIs associated to the Alzheimer's Disease (AD). Additionally, this method can be used to extract a reduced set of discriminative features for classification, since it compresses discriminative information contained in the brain. Voxels marked by ROIs which were computed using the proposed method, yield classification results up to 90% of accuracy for *controls* (CN) and Alzheimer's disease (AD) patients, and 84% of accuracy for Mild Cognitive Impairment (MCI) and AD patients.

## Introduction

Nowadays, neurodegenerative disorders affect over 30 million people around the world. Due to the increasing life expectancy and ageing of the population in developed nations, they are expected to affect 60 million people worldwide over the next 50 years. A distinctive example of these neurodegenerative disorders is the Alzheimer's Disease (AD). Indeed, the latest estimates argue for global prevalence of AD to be quadrupled by 2050. slow, degenerative disease related to pathological amyloid depositions and hyperphosphorylation of structural proteins in the brain [1]. Its progression depends on every individual, and it usually begins showing signs of mild memory problems which turn into severe brain damage some years later. As for other neurological disorders, there is currently no cure for the AD; however early diagnosis may help to slow down the rapid advance of the disease. Although the development of the disease depends on the individual, aging, etc. there are many common symptoms in addition to structural changes in the brain. Some specific image-based diagnostic methods for AD and other neurological disorders that use functional imaging have been developed previously. Since AD causes loss of brain function, affecting the areas of the brain related to memory, thought and language, it is possible to deal with automatic diagnosis tools by learning patterns associated to brain functions. In fact, there exist some methods that aim to detect functional brain variations [2]–[6] by exploiting the information contained in the images to learn patterns associated to cerebral damage.

AD can usually be diagnosed by means of neuropsychological tests [7], but at an early stage of the disease, when symptomatic cognitive problems are perceptible, a noticeable neurodegeneration has already occurred [8]. However [9], [10], suggest that pathological manifestations of AD can be detected some years earlier, before cognitive problems arise. This would allow to start specific therapy for the patient at early stages of the disease. In fact, works such as [1], [8], [11]–[14] are focused in the use of both different statistical and artificial intelligence methods to reveal patterns in MCI patients relating to either structural artrophy in MRI or functional patterns that allow to differentiate them from AD or control patients (CN). The most functional imaging-based techniques use Single Emission Computed Tomography (SPECT) [2], [3], [15] or Positron Emission Tomography (PET) images [4], [16] to detect decreased blood perfusion or decreased glucose metabolism in brain areas associated to AD. These methods aim to build models from a set of labelled images to be used for further classification of new patients. In this way, Gaussian mixture models (GMM) are used in [4], [17] to model functional images in order to select regions of interest (ROIs) for AD. Unfortunately, GMM-based methods are not straightforward to apply for ROI computation in MRI due to the high number of voxels and the high number of Gaussian components to be estimated. Consequently, it may result unfeasible in practice. See [18] for a comprehensive explanation of the maximum likelihood method and a performance comparison with other existing methods. That work concludes that the maximum likelihood estimates are the most accurate, closely followed by the regression-type estimates, quantile method, and finally, the method of moments. The only disadvantage of the maximum likelihood estimation is that this technique is most computationally expensive.

Other works such as [19], [20] use principal component analysis to extract relevant features for AD diagnosis linked to discriminative regions, though. Similarly [21], defines association rules over discriminative regions. Nevertheless, such works use functional images to reveal discriminative regions and to classify brain images.

Neurodegeneration relating to AD produces abnormal structural changes in the brain, which eventually result on extreme shrinkage of the hippocampal volume or extreme reduction of its cortical thickness, as well as a severe enlargement of the internal ventricles. However, some structural changes associated to early AD are revealed to be similar to those caused by natural ageing process. Therefore, despite AD diagnosis can be addressed by detecting these structural changes [22]–[27], it is not straightforward to distinguish them in early stages of the disease, when only minor symptomatic cognitive dysfunctions are evidenced (i.e. progressive MCI patients) [28]. A review of different methods for automatic classification of AD using MRI images from the ADNI database can be found in [13].

Unlike methods that use intensity voxels for classification [29], most methods based on MRI begin with an image segmentation into different tissues and, later, a comparison of tissue distribution. Thus [30], uses an ensemble of sparse classifiers directly over the segmented tissues [30], without parcelling ROIs. Other works, however, take into account the tissue distribution in relevant brain regions related to AD. These regions, namely Regions of Interest (ROI), can be obtained by grouping voxels into anatomical regions using a labelled atlas [13], [31], [32]. ROI-based methods such as [33] parcel the brain into ROIs or segment the hippocampus [34], [35] by wrapping an anatomy atlas, in order to compare tissue volumes in CN/AD or MCI/AD patients. These works report classification accuracies up to 90% on diagnosed AD patients through post-mortem analysis. Thus, as ROIs reveal brain areas linked to neurodegeneration, selection of voxels belonging to ROIs plays an important role in the classification task by allowing the classifier to be fed with the most discriminative information. Therefore, extracting relevant ROIs from training images that can be treated as markers, can finally determine the classification results.

Current trend on studies focused on AD/CN and MCI/AD classification consist on using a single imaging modality. As shown above, some methods use functional imaging whereas other use structural MRI, and the analysis of each image modality can reveal different markers associated to the disease. For instance, MRI analysis reports grey matter atrophy that usually results in differences in both, the hippocampus and the entorhinal cortex. Additionally, it has been proved that early changes in the hippocampal volume and entorhinal cortex are related to evidences of early AD, while this is not so obvious in other cerebral structures [1], [36]. In fact, our contribution shows that incorporating patterns revealed from differences in white matter helps to achieve better classification results.

Previous works such as [37] use MRIs from each class instead of segmented images in order to compute ROIs. On the other hand, in [37], class prototypes are computed by using the LVQ algorithm [38] and new images are projected onto these prototypes to extract features in a similar way to the *eigenface* approach [39]. Besides, in [24], a method to extract ROIs from MRIs avoiding segmentation is shown. In this case it is not necessary to perform a MRI segmentation; conversely, image preprocessing such as intensity normalization must be carried out. However, the level of accuracy provided by this method is significantly lower than the one provided by our approach, especially for MCI/AD classification, since it deals with early AD diagnosis.

**Figure 1:**
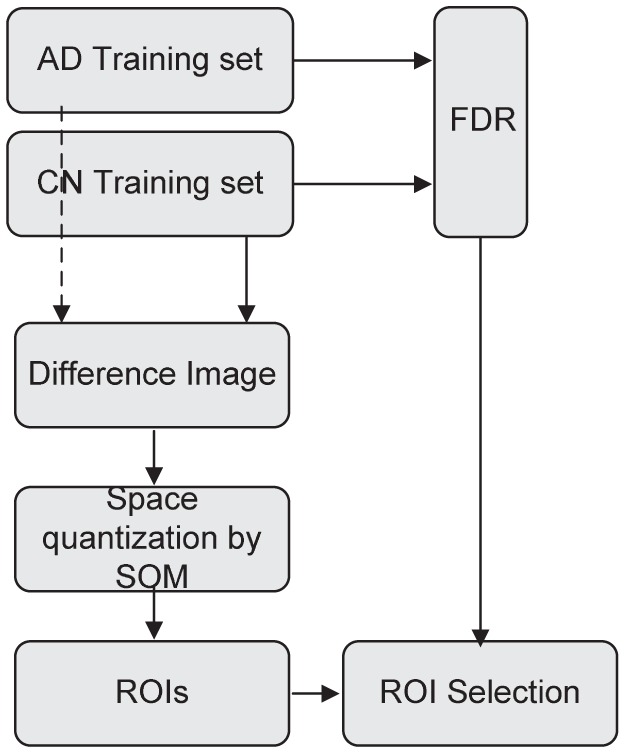
Block diagram of the proposed method for extracting and selecting ROIs.

In our work, structural MRI is used to automatically reveal 3D brain regions associated to AD by means of analyzing the distribution of WM and GM tissues in the brain. The computed ROIs are later used as a mask in order to extract voxels from relevant regions for subsequent analyses. Thus, regions linked to a specific brain disorder are automatically computed by quantizing the space into a number of regions that group similar voxels. This way, it is not necessary to get additional information from any anatomy atlas. Additionally, a measurement of relative importance is calculated for each ROI on the basis of its discriminative capability for AD diagnosis. This is accomplished by computing a number of prototype vectors using the SOM algorithm that quantize the space taking into account differences between CN and AD images. Consequently, the receptive field of each SOM unit represents a ROI on the image and its relative importance can be computed by means of the discriminative power of each voxel. Figure 1 shows the block diagram of the proposed method to extract and to selecting ROIs. The devised method has been evaluated in terms of its ability to classify new patients (not used in the training stage) correctly, through extensive experiments performed over the 1075-T1 ADNI database.

The main contributions of this work can be summarized in the following:

1. A method for automatic ROI delineation is proposed, using SOM to quantize the feature space composed by voxel coordinates and information regarding voxel differences between classes.
2. It automatically reveals brain areas (ROIs) according to patterns learnt to differentiate CN and AD patients (or MCI/AD patients).
3. A relative importance measurement is also computed for each ROI, to indicate its discriminative capability.
4. The method has been assessed through extensive experiments using 818 images from the 1075-T1 ADNI database, yielding 90% (AUC 0.92) and 84% (AUC 0.84) of classification accuracy within CN/AD and MCI/AD, respectively.

The rest of this paper is organized as follows. Section materials and methods shows the materials and methods used in this work, including the necessary background to support the next sections and details on the database used to assess the proposed method. Examples of ROI computation are also provided in this section. Experimental results section provides classification results using ROIs computed with the proposed method over the database previously described. Finally, conclusions are drawn in the conclusions section.

## Materials and Methods

### Database

Data used in the preparation of this article were obtained from the Alzheimers Disease Neuroimaging Initiative (ADNI) database (adni.loni.usc.edu). The ADNI was launched in 2003 by the National Institute on Aging (NIA), the National Institute of Biomedical Imaging and Bioengineering (NIBIB), the Food and Drug Administration (FDA), private pharmaceutical companies and non-profit organizations, as a $60 million, 5-year public-private partnership. The primary goal of ADNI has been to test whether serial magnetic resonance imaging (MRI), positron emission tomography (PET), other biological markers, and clinical and neuropsychological assessment can be combined to measure the progression of mild cognitive impairment (MCI) and early Alzheimers disease (AD). Determination of sensitive and specific markers of very early AD progression is intended to aid researchers and clinicians to develop new treatments and monitor their effectiveness, as well as lessen the time and cost of clinical trials. The Principal Investigator of this initiative is Michael W. Weiner, MD, VA Medical Center and University of California - San Francisco. ADNI is the result of efforts of many co-investigators from a broad range of academic institutions and private corporations, and subjects have been recruited from over 50 sites across the U.S. and Canada. The initial goal of ADNI was to recruit 800 subjects but ADNI has been followed by ADNI-GO and ADNI-2. To date these three protocols have recruited over 1500 adults, ages 55 to 90, to participate in the research, consisting of cognitively normal older individuals, people with early or late MCI, and people with early AD. The follow up duration of each group is specified in the protocols for ADNI-1, ADNI-2 and ADNI-GO. Subjects originally recruited for ADNI-1 and ADNI-GO had the option to be followed in ADNI-2. For up-to-date information, see www.adni-info.org.

ADNI database [40] was created to study Alzheimer disease's progression, collecting a vast amount of MRI and Positron Emission Tomography (PET) images as well as blood biomarkers and cerebrospinal fluid analyses. The main goal of this database was to provide a way to diagnose early AD stages. ADNI database provides data for three groups of subjects: healthy individuals (Controls, CN), Alzheimer disease patients (AD) and patients suffering from mild cognitive impairment symptoms (MCI). The database that has been used in this work, contains 1075 T1-weighted MRI images, comprising 229 CN, 401 MCI (312 stable MCI and 86 progressive MCI) and 188 AD images. Specifically, we have used the database *ADNI1:Screening 1.5T (subjects who have a screening data)*. This database contains MRI data from 818 subjects and repeated scans in some cases. When multiple scans of the same subject were available, the first one was selected. As a result, 818 images have been used for assessing our approach. Demographic data of patients in the database is summarized in Table 1.

**Table 1:** Demographic data of patients in the database (ADNI 1075-T1).

Diagnosis	Number	Age	Gender (M/F)	MMSE
CN	229	75.97±5.0	119/110	29.00±1.0
MCI	401	74.85±7.4	258/143	27.01±1.8
AD	188	75.36±7.5	99/89	23.28±2.0

### Image preprocessing and co-registration

Image data pre-processing, segmentation and co-registration of T1-weighted MRI images from the ADNI database have been performed. Initially, images from the ADNI database were nor skull-stripped neither spatially normalized. Thus, all the images had to be pre-processed and co-registered before segmentation. The whole process has been performed using the VBM [41] toolbox for SPM. Pre-processing, co-registration and segmentation procedures as well as the parameters used at each stage can be summarized as follows:

1. Pre-processing and co-registration.
  - The whole process was guided by means of tissue probability maps. A nonlinear deformation field that best overlays the tissue probability maps on the individual sujects' image is estimated. The tissue probability maps provided by the International Consortium for Brain Mapping (ICBM) are derived from 452 T1-weighted scans, which were aligned with an atlas space, then corrected for scan inhomogeneities, and finally classified into grey matter, white matter and cerebrospinal fluid. The data were affine registered to the MNI space and down-sampled to 2 mm resolution. Moreover, all images from the database were resized to 121×145×121 voxels.
  - A mutual information affine registration with the tissue probability maps was used to achieve approximate alignment.
  - Spatial normalization was based on a high-dimensional Dartel normalization and used standard Dartel template provided by VBM 8.
2. Segmentation.
  - The number of Gaussians used to represent the intensity distribution for each tissue class was set to 2 for all grey matter, white matter and cerebrospinal fluid. The use of multiple components per tissue allows to reckon partial volume effects and deep GM differing from cortical GM.
  - A very light bias regularization was performed to correct smooth, spatially varying artifacts that modulates the intensity of the images.
  - Gaussian bias-smoothing was not used, though, since according to our experiments, non-smoothed images provided a better classification results.
  - Warping regularization was set to 4 to determine the tradeoff between the two terms of the objective function for registering the tissue probability maps to the image to be processed. One term gives a function of how probable the data is given the parameters. The other is a function of how probable the parameters are, and provides a penalty for unlikely deformations.
  - A spatial adaptive non local means denoising filter is applied to the data in order to remove noise while preserving edges. The smoothing filter size is automatically estimated based on the local variance in the image.
  - A hidden Markov Random Field (MRF) with a weighting of 0.15 was used to encode spatial constraints of neighboring voxels. Neighboring voxels were expected to have the same class labels. The prior probability of the class and the likelihood probability of the observation were combined to estimate the Maximum a posteriori (MAP).
  - Skull stripping was performed by using SPM-VBM tool and VBM templates.

As a result, probability maps were obtained for each MRI in the database, which consisted of values in the range 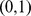 for each voxel and related to its membership probability (WM, GM or CSF tissues). However, CSF distribution was not used in our experiments.

### Background in SOM

The Self-Organizing Map (SOM) [38] is a well-known, peculiar clustering algorithm, inspired in the animal brain which seeks for the most representative and most economic representation of data and its corresponding relationships [38], [42]. During the training stage, the prototypes keep the most representative part of the input data, while the units on the output space (i.e. 2D or 3D lattice) holding similar prototypes (in terms of Euclidean distance) are moved closed together into a group. Thus, some important features of the input space can be inferred from the output space [42], regarding the input space modelling, density distribution of the data space and feature selection. SOM training is performed in a competitive way so that just a single neuron wins (i.e. its prototype vector is the most similar one to the input data instance). The most similar prototype to the input data sample is called *Best Matching Unit* (BMU) and it is computed as:
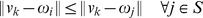
(1)where 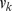 is the 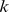-sample from the input space, 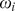 is the 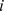-prototype (i.e. the weight associated the the 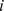-unit) and 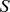 is the output SOM space.

Moreover, prototypes of neurons belonging to the neighbourhood of the BMU are also updated according to
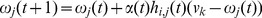
(2)where 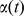 is the learning factor and 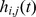 is the neighbourhood function defining the unit surrounding the BMU 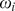.


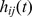
 is a Gaussian neighbourhood function 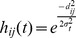, that determines the units to be updated at current iteration, where 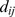 is the distance between map units 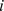 and 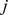 and 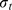 is the neighbourhood radius at time 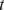. The learning rate follows the reciprocally-decreasing function 
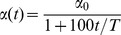
, where 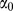 is the initial learning rate and 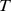 is the training length.

SOM training is accomplished in two phases. The first one provides a rough organization of the map by setting the initial radius to 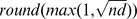 (where 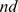 is the number of SOM units) and the initial learning rate 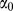 to 0.1. The second phase aims to fine-tune the map, and uses a smaller initial neighbourhood radius, specifically 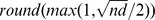. Moreover, the learning rate is also smaller in this phase, specifically 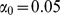.

The training process generates the prototype vectors 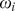 which quantize the data manifold and represent cluster centres on the data, mapped to each BMU. As SOM units are located at different positions in a 3D lattice, the feature space is projected onto a 3D space.

### Statistical significance of voxels

A MRI is composed of a high number of voxels according to a previously-stated image resolution. Images in the ADNI database used in this work contain 121×145×121 voxels after normalization. Since not all the voxels result equally discriminative for classification [21], they could be ranked according to a specific discriminative criterion. Thus, the *Fisher Discriminant Ratio* (FDR) [43] which is characterized by its separation capability as shown in [43], [44] has been used to compute the discriminative power of each voxel. In the two-class separation case, FDR is defined as
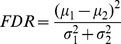
(3)where 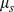 and 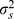 are the mean and variance values of each input variable belonging to class 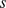, respectively. In our case, 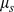 represents the mean image computed by averaging the intensity of the voxels in each definite position 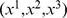 for all the images belonging to class 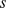 in the training set. Similarly, 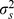 represents the variance image computed by taking into account the intensity of the voxels in a specific position 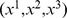 for each image belonging to class 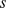 in the training set. Mean and variance images for class 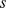 can be computed as:
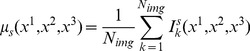
(4)where 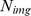 is the number of images in the training subset and 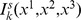 is the intensity at position 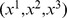 for the image 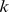.

Similarly, the variance image is defined as
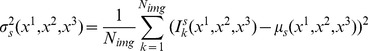
(5)From equations 4 and 5, a FDR image which contains the corresponding FDR value at each precise voxel position can be defined.

FDR value increases as the variable results in more discriminative values between the two classes. FDR values are further incorporated to the ROI computation stage to calculate the relative importance of each ROI. At this point, it is important to highlight that FDR is considered a part of the training stage. Thus, it is computed by using only images from the training subsets during cross-validation, ensuring that test data is never used for training.

**Figure 2:**
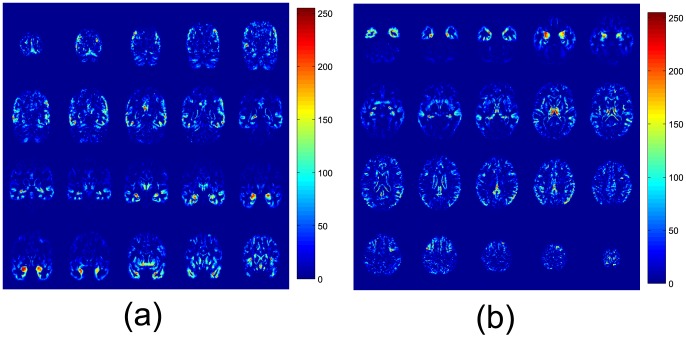
FDR values for some relevant slices on the (a) coronal and (b) axial planes for CN and AD subjects.

Figure 2 shows the FDR values computed for some relevant slices on the coronal and axial planes, according to the scale shown in the colorbar. In these figures relevant areas related to the Alzheimer's disease such as the hippocampus appear with a high FDR value, indicating a higher discriminative power over other areas.

### Feature generation and ROI modelling

Due to the high number of voxels present in a MR image, methods that aim to compress or synthesise the information contained in these voxels in a reduced number of features allow the diminishing of the classification task's computational cost. Regardless of this process, non-relevant features can be taken out. One of the classical tools for dimensionality reduction is the well-known Principal Component Analysis (PCA) [43]. PCA can be used to generate a reduced number of features from the MRI voxels in order to improve both, classification accuracy and computational effectiveness. In this paper we present a new approach that generates ROIs by quantizing the MRI space by using a SOM, and results using PCA and Voxel as Features (VAF) methods are provided as baselines for comparison.

#### Principal Component Analysis

PCA generates an orthonormal basis vector indicating the maximum variance directions. Thus, the projection onto this basis maximizes the scatter of all the projected samples. The PCA method can be briefly described as follows. Let 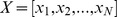 be the sample set of training vectors, where 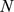 is the number of patients in the MRI database. Thus, each image is converted into a column vector that comprises the intensities of each voxel. After normalizing 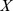 to zero mean and unity variance, we obtain a new *whithened* dataset 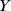 [43] and its covariance matrix can be computed as 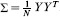. The eigenvectors 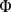 and eigenvalues 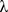 of 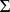 can be computed by solving 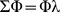. Thus, the eigenvectors or principal components (PCs) of the covariance matrix, define the directions of maximum variance of the data manifold. Usually, the eigenvectors are chosen in increasing-variance order, in such a way that the first eigenvectors compress the most part of the variance explained [43]. Subsequently, the projections of the data samples onto the low dimensional space spanned by the principal components are used in the classification task.

#### ROI modelling using SOM

The method presented in this paper aims not only to reduce the dimensionality of the input space but also to compute ROIs and then weight them according to their relative importance. The core idea of the method consists on quantize the MRI space by a number of prototypes which model the intensity distribution in different brain areas. Thus, information about intensity is incorporated to the quantization process in order to group the voxels according to their intensity level and being later capable to model the MRI intensity distribution in the SOM space. Therefore, the feature space is composed by vectors which contain the voxel's three-coordinates location in the three first coordinates and the intensity difference between CN and AD images. Such a difference image is computed as
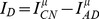
(6)where 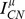 and 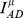 are the mean image of normal and AD images, respectively.

**Figure 3:**
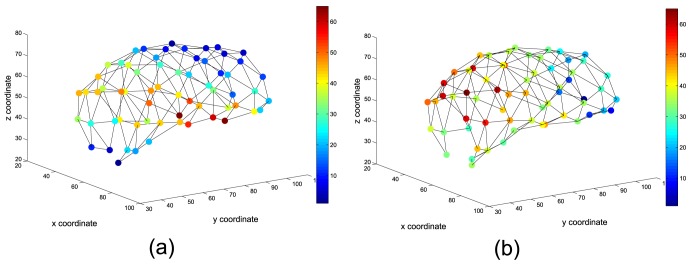
SOM units after training. The position of each unit represent the cluster centre in the brain, indicating areas with similar intensities. Intensity difference associated to each cluster is indicated using colours according to the colorbar. (a) and (b) show the model for GM and WM respectively.

The feature space to be quantized is then formed by vectors 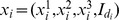, where 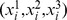 are the coordinates of the voxel 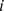 (i.e. 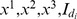 are components of 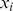) and 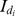 is the intensity of voxel 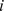 in the difference image. Prototypes computed by means of vector quantization are cluster centres and represent groups of similar instances according to the Euclidean distance in the feature space (voxels), namely Receptive Field (RF). From the BMU concept described in section, the RF of a unit 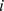 (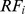) can be defined as
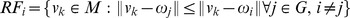
(7)denoting the set of input data vectors for which 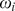 is the BMU. Subsequently, the relative importance of each ROI is computed according to the following equation
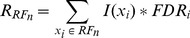
(8)where 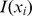 and 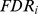 denote the intensity and the FDR value respectively, of the voxel at coordinates 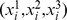. Figure 3 shows the SOM units after training when using a 3D cylindrical (infinite plane) lattice [38]. The first three coordinates of the SOM prototypes determine the position of each unit which represents a cluster centre. The fourth coordinate is depicted using different colours indicating the importance of the ROI according to the colorbar.

The core idea behind the presented method consists on quantizing the space to a number of model vectors, and to categorize them according to their relative importance and the level of intensity in the difference image. Consequently, the number of extracted ROIs corresponds to the number of units in the SOM, and the size of each ROI depends on the size of the receptive field of the corresponding SOM unit.

The self-organizing process carried out by SOM provides the models shown in Figure 3, where each unit represents a cluster centre in the brain, indicating areas with similar intensities in the difference image. Thus, models for GM and WM are built as shown in Figures 3(a) and 3(b) respectively. As previously indicated, feature vectors used as inputs for the SOM are 4-dimensional. The fourth dimension in the feature space is the intensity difference for each voxel, indicated using a colour code in the prototypes according to the colour bar at the right side in the figures. Images from CN or AD patients can be projected onto the SOM space showing the relative importance of each unit (that indicates ROI centre) by means of 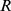 value computed according to equation 8.

**Figure 4:**
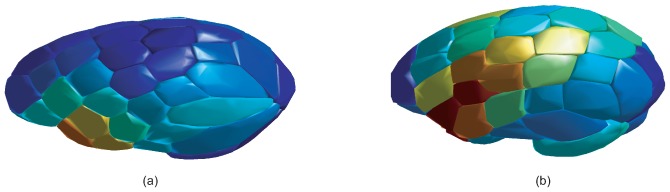
3D reconstruction from ROIs computed using the SOM model for (a) GM and (b) WM tissues.

A 3D volume can be reconstructed from the SOM model by means of the vector quantization process as depicted in Figure 4, where the RFs of SOM units define the size of each ROI. In this figure, 3D models for GM (a) and WM (b) are shown.

**Figure 5:**
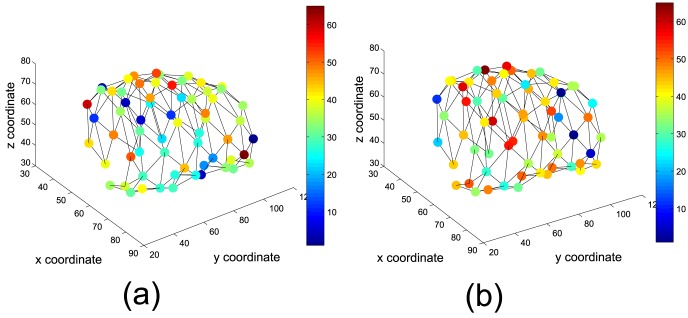
Projection of images in the SOM GM model for (a) CN and (b) AD example patients from the database. Units indicate the position of ROI centres and 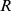 value is encoded according to the corresponding colour bar.

Figures 5(a) and 5(b) show the position of the ROI centres for CN and AD patients respectively and units are coloured according to its 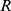 value. In these figures, different patterns associated to CN and AD are revealed and they are clearly shown.

**Figure 6:**
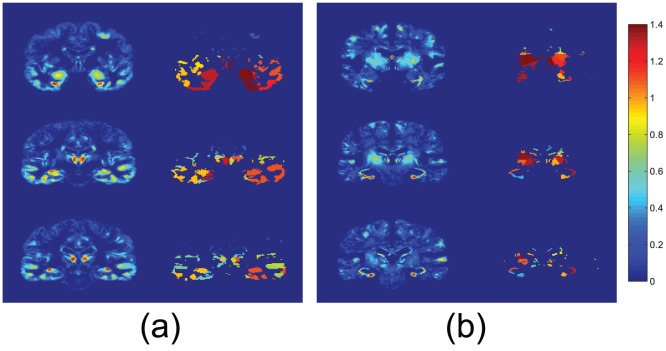
Difference image (right column) and ROIs computed by the proposed method (left) for (a) GM and (b) WM, respectively for CN/NOR images. Images from a random training subset from the cross-validation folds have been used. ROIs are coloured according to the colorbar scale due to their relative importance.

Figures 6(a) and 6(b) show the difference between CN and AD images in the left column and the ROIs computed in the right column. Each ROI is marked with a different colour that indicates the relevance of that ROI according to its discriminative power. ROIs depicted in Figure 6 have been computed using FDR values above 90% of its maximum value.

**Figure 7:**
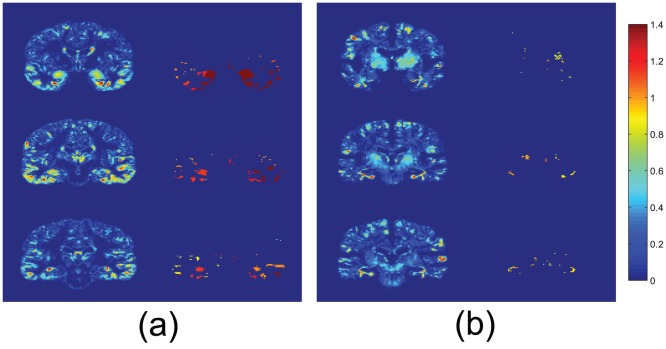
Difference image (right column) and ROIs computed by the proposed method (left) for (a) GM and (b) WM, respectively, for MCI/AD images. Images from a random training subset from the cross-validation folds have been used. ROIs are coloured according to the colorbar scale due to its relative importance.

Similarly, Figures 7(a) and 7(b) show difference images and ROIs coloured according to its discriminative power for MCI/AD images. It is worth mentioning that most relevant ROIs computed by the proposed method, specially in GM, are compatible with areas that appear in literature as representative regions of AD, located in the temporal lobe, such as hippocampus and the superior temporal gyrus which are responsible for the individual's memory formation, speech perception, and language skills [45], [46]. In the case of MCI patients, the method truly reveals structural changes mainly focused on the hippocampus area.

### Classification using Support Vector Machine (SVM)

Classification of the feature vectors consisting of the relative importance measure computed as indicated in the ROI modelling section is accomplished by means of Support Vector Machine (SVM). SVMs were introduced in 70's by Vapnik [47] and consisted of a set of supervised learning methods widely used for classification and regression [47], [48], which were designed to split off a set of binary-labelled data by means of a hyperplane. Specifically, they compute the maximal margin hyperplane to achieve maximum separation between classes. SVMs operate by building a decision function in the form 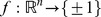 using 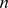-dimensional training vectors 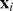 and class labels 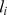:
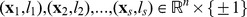
(9)in such a way that 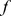 is able to correctly classify new samples 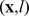. Linear discriminative functions define decision hyperplanes in a multidimensional feature space:
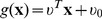
(10)where 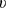 is the weight vector and 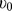 is a bias (threshold). This way, 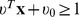 if class 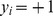 and 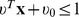 if class 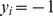, and the weight vector 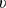 is orthogonal to the decision hyperplane. Finding the optimal separating hyperplane is accomplished by an optimization task consisting of finding the unknown parameters 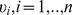 which define the decision hyperplane. Let 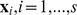 be the feature vectors of the training set 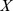. These belong to one of the two classes, either 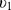 or 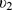. If the classes are linearly separable, the objective would be to design a hyperplane that correctly classified all the training vectors. As a result, that hyperplane is not unique and the optimization process focuses on maximizing the generalization performance of the classifier, which can be defined as the effectiveness of the classifier to operate with new data. Among some other criteria, the maximal margin hyperplane is usually selected since it provides the maximum margin of separation between the two classes. Since the distance from a point 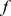 to the hyperplane is given by 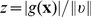, to scale 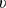 and 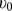 so that the value of 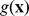 is 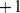 for the nearest point in 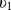 and 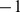 for the nearest points in 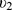, reduces the optimization problem to maximizing the margin 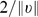 with the constrains:
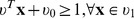
(11)

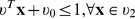
(12)Thus, designing the classifier leads to a non-linear (quadratic) optimization task subject to a set of linear inequality constrains. The solution 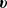 is found to be a linear combination of 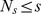 feature vectors and the optimum hyperplane is called the *support vector machine*.

For non-linearly separable data, the optimization process needs to be modified to work in combination with kernel techniques, so that the hyperplane that defines the SVM corresponds to a non-linear decision boundary in the input space. The use of kernels enables to map the data into some other dot product space 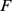 (namely, feature space) through a non-linear transformation 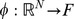, and perform the described linear algorithm in 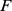. Thus, the decision function is nonlinear in the input space and takes the form
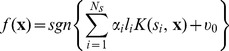
(13)where parameters 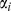 and 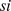 are the solution for the optimization process, solved by either Quadratic Programming (QP) or the well-known Sequential Minimal Optimization (SMO) [49] and the support vectors [47] (i.e. the training vectors that are closest to the linear classifier since lie on either of the two hyperplanes, i.e. 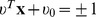. In our case, we used a Radial Basis Function (RBF) 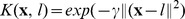 as kernel function. In fact, the use of radial basis functions as kernels in SVMs have proved to supply better results than linear kernels for different applications [11], [50].

## Results and Discussion

**Figure 8:**
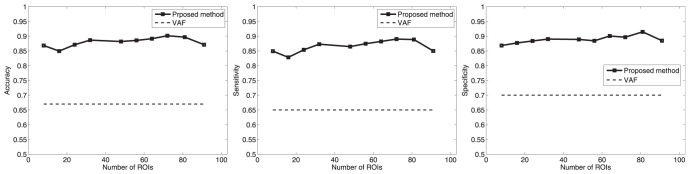
Classification results for CN/AD.

**Figure 9:**
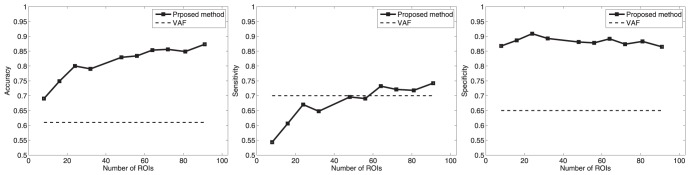
Classification results for MCI/AD.

To demonstrate the relevance of the proposed ROI selection method for diagnosis purposes, we applied the devised ROI selection algorithm to the overall database described in the *Database* subsection, including CN, MCI and AD patients. Such a procedure aims to objectively evaluate the discriminative power of the computed ROIs. Thus, classification through SVM supervised learning is carried out to group the images according to the value of 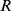 obtained in equation 8 for each ROI. All the classification experiments were performed considering different subsets for cross validation. Specifically, sets of CN/AD and MCI/AD patients were obtained from the data manifold using *k-fold*, which takes 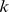 images for testing and use the rest for training. As usual, we used 10-fold (k = 10) in our experiments. Results for CN/AD and MCI/AD classification are shown in Figures 8 and 9, respectively. These results were compared to the Voxels As Features (VAF) technique [5], [51] which uses all the intensity voxels to feed the classifier in both, training and testing stages. In other words, VAF does not concern any feature generation or selection technique. On the contrary, scaled versions of the probability maps obtained as indicated in the *Image Preprocessing* section from segmented images, were used as features. Since VAF uses all the information contained in the images without selecting discriminative voxels, it may decrease the classification performance. Our method, which uses voxel selection and feature generation associated to ROIs, outperforms the VAF approach as shown in Figure 8. Additionally, experiments using PCA as described in the *Statistical significance of voxels* section, were performed for comparison. Our proposed method also outperforms the approach which uses PCA for feature extraction as shown in Table 2 as well. The experiments described above state that computed ROIs effectively indicate brain areas concerning AD, as above described. In fact, hippocampus and primary auditory cortex areas are marked as relevant regions in the computed ROIs. We also found that ventricles were marked too, which would correspond to brain areas as indicated in the literature [45], [46], [52] affected by atrophy process linked to neurodegeneration in AD.

**Table 2:** Classification results for different voxel selection methods.

Measure	Proposed method	PCA-SVM	VAF-SVM
*CN/AD*			
Accuracy	0.90±0.06	0.82±0.04	0.67±0.04
Sensitivity	0.87±0.07	0.80±0.08	0.65±0.08
Specificity	0.92±0.09	0.84±0.06	0.70±0.05
*MCI/AD*			
Accuracy	0.83±0.06	0.70±0.06	0.61±0.07
Sensitivity	0.82±0.07	0.61±0.08	0.60±0.08
Specificity	0.87±0.09	0.72±0.10	0.75±0.09

Similarly to the experiments described above for CN/AD patients, experiments that aim to differentiate between MCI and AD patients were carried out. Thus, MCI/AD classification results are shown in Figure 9. In this case, as image differences between MCI and AD are not so evident as in the CN/AD case, specific feature generation and reduction methods may result especially relevant to improve classification outcomes. Moreover, brain areas revealed through differences between MCI and AD patients are not as clear as in the CN/AD case, and ROI selection helps figure out patterns relating to MCI. In this way, computed ROIs show differences mainly focused in the hippocampus area, stating differences associated to the atrophy in this brain area.

At the same time, ROIs extracted from MCI patients have been assessed by means of classification experiments. Figures 8 and 9 shows the performance of our method, which clearly outperforms the VAF approach. Moreover, these results set the stability in terms of the number of SOM prototypes of the proposed approach to compute ROIs and their relevance for classification.

**Figure 10:**
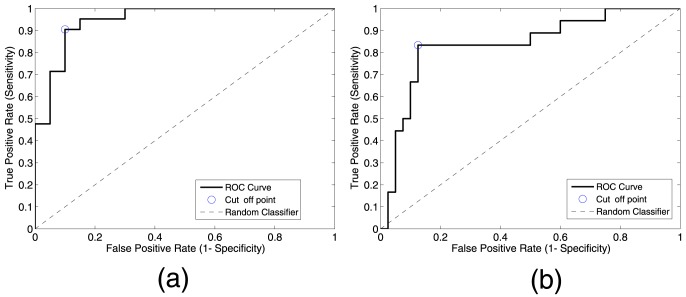
ROC curves for (a) CN/AD and (b) MCI/AD classification, respectively.

Figure 8 also states the effectiveness of the proposed method for extracting relevant brain areas relating to to AD, and the assignment of a relative relevance value to each one of these regions. Thus, accuracy values up to 90% and sensitivity values up to 87% were obtained, outperforming the classification results obtained when features are computed by means of classical techniques such as PCA. On the other hand, classification results between MCI and AD are shown in Figure 9, providing 83% of accuracy and 76% of sensitivity levels. Moreover, Figure 10(a) shows the Receiving Operating Curve (ROC) for MCI/AD classification, yielding an Area Under ROC curve (AUC) of 0.92. Similarly, ROC curve for MCI/AD is depicted in Figure 10(b), where the computed AUC was 0.84, indicating high sensitivity for AD patients.

It is worth noting that there are several factors that may affect the classification results. One of them is related to the *gold-standard* diagnosis in the ADNI database, as only living subjects were analysed. This fact is specially relevant due to the difficulty for diagnosing AD *in vivo* [8], [51]. In fact [51], shows that AD classification accuracy level diminishes when autopsy data is not available. In other words, patients whose medical records are contained in the ADNI database do not usually show severe AD, but mild AD symptoms [8]. This way, as results provided in this work are close to the ones obtained in other recent works such as [13]. Therefore, it can be stated that differences in MCI patients determine the effectiveness of ROI computation. Consequently, the experiments brought about within this work show the importance of the feature selection methods in AD diagnosis through MRI imaging, which are even more relevant in the case of MCI classification.

## Conclusions

In this work, a method based on unsupervised vector quantization techniques for automatic ROI calculation is presented. Specifically, a three-dimensional Self-Organizing Map is used to model MRI images selecting ROIs related to a particular neurological disorder. Images from controls and AD patients are used to compute tissue differences by means of voxel contrastation. Subsequently, the MRI space is quantized, computing a number of prototype vectors from features extracted taking into account the spatial relationship among voxels, figuring out similar areas within the difference image. Additionally, the relative importance of each cluster is computed by means of the cluster sizes and the statistical significance of the discriminative power of the voxels that make up the ROI. The spatial relationship among clusters is also preserved in the SOM output space due to the SOM's topology preserving properties. The method has been assessed by selecting a set of images from the ADNI [40] database, using the ROIs automatically computed using the presented approach. Moreover, most relevant ROIs computed across this method, specially in GM, are compatible with areas that appear in literature as representative regions of AD such as hippocampus [45], [46], [52]. The classification results provide average accuracy, sensitivity and specificity values of up to 90%, 87% and 92%, respectively, for 10 cross-validation folds, while the AUC is 0.92. On the other hand, our method is capable to distinguish MCI from AD patients up to 83% of accuracy and AUC of 0.84. Classification experiments state the effectiveness of the proposed method to select relevant brain areas that are related to Alzheimer's disease. Since structural differences between MCI and AD are not as clear as in the CN/AD case, results obtained for MCI/AD classification indicate the applicability of the method for an early diagnosis of AD. Furthermore, it is also devised to be applicable to uncover some other hidden neurological disorders, through small structural brain alterations, as well as be useful to explore adjacent brain areas related to such disorders.

## References

[1] Westman E, Simmons A, Zhang Y, Muehlboeck J, Tunnard C, et al (2011) Multivariate analysis of mri data for alzheimer's disease, mild cognitive impairment and healthy controls. Neuroimage 54: 1178–1187. 20800095 10.1016/j.neuroimage.2010.08.044

[2] Górriz J, Segovia F, Ramírez J, Lassl A, Salas-González D (2011) Gmm based spect image classification for the diagnosis of Alzheimer's disease. Applied Soft Computing 11: 2313–2325.

[3] López M, Ramírez J, Górriz J, Álvarez I, Salas-González D, et al (2011) Principal component analysis-based techniques and supervised classification schemes for the early detection of Alzheimer's disease. Neurocomputing 74 (8) 1260–1271.

[4] Segovia F, Górriz J, Ramírez J, Salas-González D, Álvarez I, et al (2012) A comparative study of the feature extraction methods for the diagnosis of Alzheimer's disease using the adni database. Neurocomputing 75: 64–71.

[5] Stoeckel J, Fung G (2005) Svm feature selection for classification of spect images of Alzheimer's disease using spatial information. In: Proc. Fifth IEEE Int Data Mining Conf. doi:10.1109/ICDM.2005.141.

[6] Spetsieris PG, Ma Y, Dhawan V, Eidelberg D (2009) Differential diagnosis of parkinsonian syndromes using pca-based functional imaging features. Neuroimage 45: 1241–1252. 19349238 10.1016/j.neuroimage.2008.12.063

[7] Albert M, Moss M, Tanzi R, Jones K (2001) Preclinical prediction of ad using neuropsychological tests. J Int Neuropsychol Soc 70: 631–639. 10.1017/s1355617701755105 11459114

[8] Hinrichs C, Singh V, Xu G, Johnson S (2011) the Alzheimers Disease NEuroimaging Initiative (2011) Predictive markers for ad in multi-modality framework: An analysis of mci progression in the adni population. Neuroimage 55: 574–589. 21146621 10.1016/j.neuroimage.2010.10.081 PMC3035743

[9] Canu E, McLaren D, Fitzgerald M, Bendlin B, Zoccatelli G, et al (2011) Microstructural diffusion changes are independent of macrostructural volume loss in moderate to severe Alzheimer's disease. J Alzheimers Dis 19. 10.3233/JAD-2010-1295 PMC2889147 20157252

[10] Thompson P, Apostolova L (2007) Computational anatomical methods as applied to ageing and dementia. Br J Radiol 800: 78–91. 10.1259/BJR/20005470 18445748

[11] Ramírez J, Górriz J, Salas-Gonzalez D, Romero A, López M, et al (In press.) Computer-aided diagnosis of alzheimer's type dementia combining support vector machines and discriminant set of features. Information Sciences

[12] Jack CJ, Shiung M, Weigand S, O'Brien P, Gunter J, et al (2005) Brain atrophy rates predict subsequent clinical conversion in normal elderly and amnestic mci. Neurology 65: 1227–1231. 16247049 10.1212/01.wnl.0000180958.22678.91 PMC2753547

[13] Cuingnet R, Gerardin E, Tessieras J, Auzias G, Lehéricy S, et al (2010) Automatic classification of patients with Alzheimer's disease from structural MRI: a comparison of ten methods using the adni database. Neuroimage 56 (2) 766–781. 20542124 10.1016/j.neuroimage.2010.06.013

[14] Minoshima A, Foster N, Kuhl D (1994) Posterior cinculate cortex in alzheimer's disease. Lancet 3440: 895. 10.1016/s0140-6736(94)92871-1 7916431

[15] Ramirez J, Chaves R, Gorriz JM, Lopez M, Alvarez IA, et al. (2009) Computer aided diagnosis of the Alzheimer's disease combining spect-based feature selection and random forest classifiers. In: Proc. IEEE Nuclear Science Symp. Conf. Record (NSS/MIC). pp.2738–2742. doi:10.1109/NSSMIC.2009.5401968.

[16] Alvarez I, Gorriz J, Ramirez J, Salas-Gonzalez D, Lopez M, et al (2011) 18f-fdg pet imaging analysis for computer aided Alzheimer's diagnosis. Information Sciences 184 (4) 903–196.

[17] Górriz J, Lassl A, Ramírez J, Salas-Gonzalez D, Puntonet C, et al (2009) Automatic Selection of ROIs in functional imaging using Gaussian Mixture Models. Neuroscience Letters 460: 108–111. 19454303 10.1016/j.neulet.2009.05.039

[18] Vecchi MP, Kirkpatrick S (1983) Global wiring by simulated annealing. 2: 215–222. 10.1126/science.220.4598.671 17813860

[19] Alvarez I, Gorriz J, Lopez MM, Perez J, Salas-Gonzalez D, et al (2011) Computer aided diagnosis of Alzheimer's disease using component based svm. Applied Soft Computing 11: 2376–2382.

[20] Álvarez I, Górriz J, Ramírez J, Salas-González D, López M, et al (2010) Projecting independent components of spect images for computer aided diagnosis of Alzheimer's disease. Pattern Recognition Letters 31 (11) 1342–1347.

[21] Chaves R, Ramírez J, Górriz J, Álvarez I (2012) Functional brain image classification using association rules defined over discriminant regions. Pattern Recognition Letters 33 (12) 1666–1672.

[22] Termenon M, Graña M (2012) A two stage sequential ensemble applied to the classification of Alzheimer's disease based on mri features. Neural Processing Letters 35 (1) 1–12.

[23] Chyzhyk D, Graña M, Savio A, Maiora J (2012) Hybrid dendritic computing with kernel-lica applied to Alzheimer's disease detection in mri. Neurocomputing 75 (1) 72–77.

[24] Ortiz A, Górriz J, Ramírez J, Martínez-Murcia F (2013) LVQ-SVM based CAD tool applied to structural MRI for the diagnosis of the Alzheimers disease. Pattern Recognition Letters 34: 1725–1733.

[25] Westman E, Muehlboeck J, Simmons A (2012) Combining mri and csf measures for classification of alzheimer's disease and prediction of mild cognitive impairment conversion. Neuroimage 62. 10.1016/j.neuroimage.2012.04.056 22580170

[26] Frisoni G, Fox N, Jack C Jr, Scheltens P, Thompson P (2010) The clinical use of structural mri in alzheimer disease. Nature Reviews Neurology 6: 67–77. 20139996 10.1038/nrneurol.2009.215 PMC2938772

[27] Bryant C, Giovanello K, Ibrahim J, Chang J, Shen D, et al (2013) Mapping the Genetic Variation of Regional Brain Volumes as Explained by All Common SNPs from the ADNI Study. PLOS ONE 8: 1–9. 10.1371/journal.pone.0071723 PMC3756017 24015190

[28] Tondelli M, Wilcock G, Nichelli P, De Jager C, Jenkinson M, et al (2012) Structural mri changes detectable up to ten years before clinical alzheimer's disease. Neurobioly Aging 33: 25–36. 10.1016/j.neurobiolaging.2011.05.018 21782287

[29] Casanova R, Hsu FC, Espeland MA (2012) Alzheimer's Disease Neuroimaging Initiative (2012) Classification of structural mri images in alzheimer's disease from the perspective of ill-posed problems. PLoS ONE 7: e44877. 23071501 10.1371/journal.pone.0044877 PMC3468621

[30] Liu M, Zhang D, Shen D (2012) for the Alzheimer's Disease Neuroimaging Initiative (2012) Ensemble sparse classification of alzheimer's disease. Neuroimage 60: 1106–1116. 22270352 10.1016/j.neuroimage.2012.01.055 PMC3303950

[31] Lao Z, Shen D, Xue Z, Karacali B, Resnick S, et al (2004) Morphological classification of brains via high-dimensional shape transformations and machine learning methods. Neuroimage 21: 46–57. 14741641 10.1016/j.neuroimage.2003.09.027

[32] Magnin B, Mesrob L, Kinkingnéhun S, Pélégrini-Issac M, Colliot O, et al (2009) Support vector machine-based classification of alzheimer's disease from whole-brain anatomical mri. Neuroradiology 51: 73–83. 18846369 10.1007/s00234-008-0463-x

[33] Desikan R, Cabral H, Hess C, Dillon W, Glastonbury C, et al (2009) Automated mri measures identify individuals with mild cognitive impairment and alzheimer's disease. Brain 132: 2048–2057. 19460794 10.1093/brain/awp123 PMC2714061

[34] Chupin M, Hammers A, Liu R, Colliot O, Burdett J, et al (2009) Automatic segmentation of the hippocampus and the amygdala driven by hybrid constraints: method and validation. Neuroimage 46: 749–761. 19236922 10.1016/j.neuroimage.2009.02.013 PMC2677639

[35] Chupin M, Gérardin E, Cuingnet R, Boutet C, Lemieux L, et al (2009) Fully automatic hippocampus segmentation and classification in alzheimer's disease and mild cognitive impairment applied on data from adni. Hippocampus 19: 579–587. 19437497 10.1002/hipo.20626 PMC2837195

[36] O'Brien J (2007) Role of imaging techniques in the diagnosis of dementia. Br J Radiol 80. 10.1259/bjr/33117326 18445747

[37] Ortiz A, Górriz J, Ramírez J, Salas-Gonzalez D (2013) Automatic roi selection using som modelling in structural brain mri. Natural and Artificial Computation in Engineering and Medical Applications Lecture Notes in Computer Science 7931: 278–285.

[38] Kohonen T (2001) Self-Organizing Maps. Springer.

[39] Turk M, Pentland A (1991) Eigenfaces for recognition. Journal of cognitive Neuroscience 3: 71–86. 23964806 10.1162/jocn.1991.3.1.71

[40] Alzheimer's Disease Neuroimaging Initiative. Available: http://adni.loni.ucla.edu/. Accessed 2014 Mar 10.

[41] Structural Brain Mapping Group Department of Psychiatry. Available: http://dbm.neuro.unijena.de/vbm8/VBM8-Manual.pdf. Accessed 2014 Mar 10.

[42] Haykin S (1999) Neural Networks. Prentice-Hall, 2nd edition.

[43] Theodoridis S, Koutroumbas K (2009) Pattern Recognition. Academic Press.

[44] Padilla P, López M, Górriz J, Ramírez J, Salas-González D, et al (2012) NMF-SVM based CAD tool applied to functional brain images for the diagnosis of Alzheimer's disease. IEEE Transactions on medical imaging 2: 207–216. 10.1109/TMI.2011.2167628 21914569

[45] Minoshima S, Giordani B, Berent S, Frey KA, Foster NL, et al (1997) Metabolic reduction in the posterior cingulate cortex in very early Alzheimer's disease. Annals of Neurology 42: 85–94. 9225689 10.1002/ana.410420114

[46] Rohkamm R (2004) Color Atlas of Neurology. Thieme, 1st edition.

[47] Vapnik VN (1998) Statistical Learning Theory. Wiley-Interscience.

[48] Sammut C, Webb GI (2010) Statistical Learning Theory. Springer.

[49] Platt J (1999) Advances in Kernel Methods - Support Vector Learning, chap. Fast Training of Support Vector MAchines using Sequential Minimal Optimization.

[50] Clarkson P, Moreno P (1999) On the use of support vector machines for phonetic classification. In: Proc. of the IEEE Int. Conference on Acoustincs, Speech and Signal Processing. volume 2, pp.585–588.

[51] Klöppel S, Stonnington CM, Chu C, Draganski B, Scahill RI, et al (2008) Automatic classification of mr scans in Alzheimer's disease. Brain 131: 681–689. 18202106 10.1093/brain/awm319 PMC2579744

[52] Mesrob L, Magnin B, Colliot O, Sarazin M, Hahn-Barma B, et al (2009) Identification of artophy patterns in alzheimer's disease based on svm feature selection and anatomical parcellation. Annals of the BMVA 2009: 1–9.

